# Prediction of blood culture outcome using hybrid neural network model based on electronic health records

**DOI:** 10.1186/s12911-020-1113-4

**Published:** 2020-07-09

**Authors:** Ming Cheng, Xiaolei Zhao, Xianfei Ding, Jianbo Gao, Shufeng Xiong, Yafeng Ren

**Affiliations:** 1 Department of Medical Information, The First Affiliated Hospital of Zhengzhou University, Zhengzhou, China; 2 Department of General ICU, The First Affiliated Hospital of Zhengzhou University, Zhengzhou, China; 3 Department of Radiology, The First Affiliated Hospital of Zhengzhou University, Zhengzhou, China; 4 School of Information Engineering, Zhengzhou University, Zhengzhou, China; 5 Computer School, Pingdingshan University, Pingdingshan, China; 6 Collaborative Innovation Center for Language Research and Services, Guangdong University of Foreign Studies, Guangzhou, China

**Keywords:** Hybrid neural network, Long short-term memory, Electronic health records, Positive blood cultures prediction

## Abstract

**Background:**

Blood cultures are often performed to detect patients who has a serious illness without infections and patients with bloodstream infections. Early positive blood culture prediction is important, as bloodstream infections may cause inflammation of the body, even organ failure or death. However, existing work mainly adopts statistical models with laboratory indicators, and fails to make full use of textual description information from EHRs.

**Methods:**

We study the problem of positive blood culture prediction by using neural network model. Specifically, we first construct dataset from raw EHRs. Then we propose a hybrid neural network which incorporates attention based Bi-directional Long Short-Term Memory and Autoencoder networks to fully capture the information in EHRs.

**Results:**

In order to evaluate the proposed method, we constructe a dataset which consists of totally 5963 patients who had one or more blood cultures tests during hospitalization. Experimental results show that the proposed neural model gets 91.23% F-measure for this task.

**Conclusions:**

The comparison results of different models demonstrated the effectiveness of our model. The proposed model outperformed traditional statistical models.

## Background

With the rapid development of computing technologies, more and more medical monitoring equipments and software systems are used in clinical practice, generating a large amount of data. This provides opportunities and challenges to accelerate clinical science using large scale of practical clinical data in less expense [1, 2]. For this reason, machine learning has been increasing impact for medical information research. Various machine learning techniques have been used to mine clinical knowledge [3–7]. Earlier work demonstrated the feasibility of building predictive models with clinical data [8, 9]. Ideally, we wish to be able to establish such models from data routinely collected in Electronic Health Records (EHRs) [10]. In the present research, our aim is to construct a novel model for predicting the risk of bloodstream infection of patients during hospitalization by predicting positive Blood Cultures (BCs).

The positive BCs is defined as a blood sample in which bacteria or fungi are present. The growth of bacterial or fungi in the blood can cause inflammation of the body, even organ failure or death [11]. When test positive is suspected blood is drawn for blood culture and the patient is started on antibiotics. On average for every culture-positive results an additional more patients receive antibiotic treatment contributing to antibiotic resistance in the community and increased healthcare costs [12]. Rapid identification of positive BCs is important for the rapid initiation of optimal treatment in patient. When BCs results are not available, the decision to continue or stop antibiotics is made based on laboratory test and the clinical profile of the patient. However patients’ clinical descriptions are complex unstructured texts and not fully understood [13, 14].

In recent years, some researchers have noticed the importance of these problems [15, 16]. Matheny et al. [17] developed a hybrid rules and natural language processing methods for detection of blood culture bacterial contamination. Steenkiste et al. [18] proposed a temporal computational model to explore for the potential prediction of the outcome of a blood culture test based on nine clinical parameters measured over time. However, this model only uses the numerical physical indicators.

**Fig. 1:**
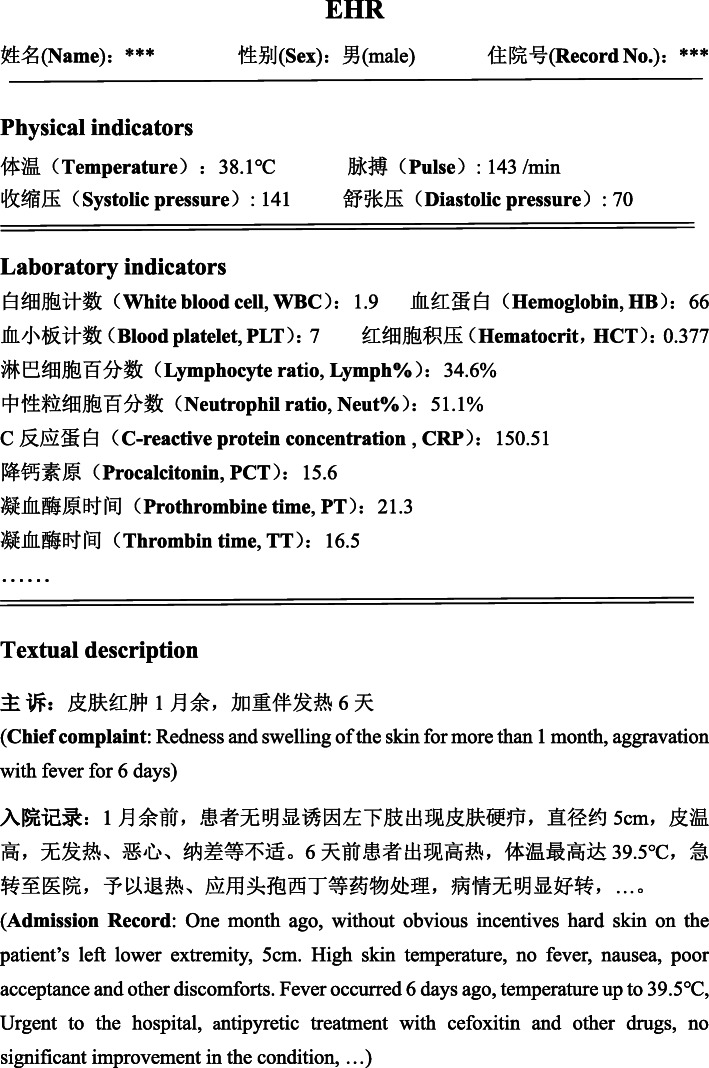
A piece of EHRs from a patient
- The hybrid model incorporates Attention-based Bi-directional Long Short Term Memory (ABiLSTM) and Denoising Autoencoder (DAE) network. The ABiLSTM is used to extract textual features and DAE takes the numerical indicators as input for capturing important numerical features.
- Conduct an extensive and large-scale empirical study to evaluate the effectiveness of the our method.

Motivated by these observation, we propose a novel hybrid neural network model which could extract the laboratory and the clinical description features simultaneously, for predicting positive blood culture based on EHRs. Electronic Health records usually contain two main information: textual description and discrete laboratory physical indicators. A piece of EHRs are shown in Fig. 1. We can see a patient’s Chief Complaints (CC), Admissions Records (AR), physical and laboratory indicators. The main contributions of the proposed method can be summaried as follows:

- In the study, we construct a dataset from a large amount of raw EHRs which contained one or more blood culture tests taken during hospitalization.

## Related work

There are a number of studies that use machine learning techniques in the field of disease prediction [19, 20]. The majority of these works focused on the numerical factors including physical examination factors and laboratory indicators. For example, Zou et al. [21] used decision tree, random forest and neural network to predict diabetes mellitus based on 14 clinical attributes. Ding et al. [22] applied a random forest model for predicting acute respiratory distress syndrome events in ICU patients based on 42 clinical variables. Yin et al. [23] used preprocedural clinical variables to develop a model for prediction of contrast-induced nephropathy (CIN) before radiological procedures among patients administered contrast media.

Moreover, some researches have conducted to the early detection of bloodstream infecions by predicting the outcome of blood cultures [18, 24]. Mani et al. [25] developed non-invasive predictive models for late onset neonatal sepsis based on the electronic medical records. A blood culture was taken to further differentiate between negative and positive culture sepsis. Instead of exclusively looking at physiological features, Lukaszewski et al. [26] trained an artificial neural network model to predict positive BCs. This model could correctly predict the outcome of the blood culture test in 83.09% of patient case. However, this research was performed based a limited data set of only 92 patients.

Previous researches mainly uses clinical laboratory parameters to predict diseases without directly taking into account unstructureal clinical description from the EHRs. Here, we present a hybrid neural network model which could extract the laboratory and the clinical description features simultaneously from EHRs to predict the outcome of blood cultures. The models may contribute to the discontinuation of antibiotics in negative cases before BCs results become available. The end results could be reduced antibiotic use with its associated benefits for the patient and for healthcare utilization. To this end, we explore and illustrate the potential of neural networks in the accurate prediction of positive blood cultures.

## Methods

### Task modeling

When doctors suspect a patient to test positive they can decide to advance to a blood culture test, the task aims to construct a model to predict positive blood culture results. We model the prediction task based on the following steps.

- We construct a dataset *D*^∗^ from the real EHRs dataset. Specifically, positive examples indicate that patients have positive blood culture results at least once during hospitalization, which is denoted as *D*_+_∈*D*^∗^. Negative examples indicate that the result of patient’s blood culture were all negative, which is denoted as *D*_−_∈*D*^∗^.
- At the training phase, we use the data *D*^∗^ that contains both *D*_+_ and *D*_−_ to train our model *M*.
- At the test phase, we apply the well-trained model *M* to predict patient’s blood culture test result, which can distinguish patients who has a serious illness without infections or patients with bloodstream infections.

### Hybrid neural network model

**Fig. 2:**
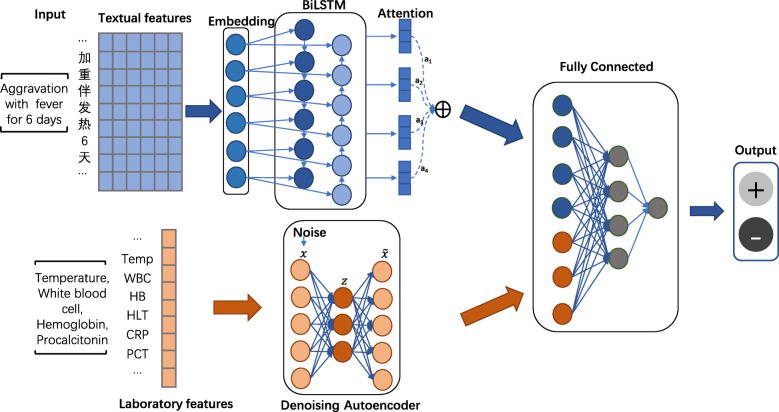
The proposed hybrid neural network framework

Our proposed hybrid neural network model including two main parts: attention-based BiLSTM and Autoencoder, the whole architecture of our method can be found in Fig. 2. ABiLSTM is used for learning continuous representation from the textual description information. The textual information contain the patient’s chief complaints and admissions records in EHRs. Chief complaints refer to patient’s symptoms and feeling of abnormal physiological function when the patient is ill. The admissions records are the text description of patient’s present illness at admission. Plenty of useful information is embedded in clinical text, which are critical for disease analysis [27, 28]. In addition, the Autoencoder is used to learn continuous representation from the laboratory biochemical indicators in EHRs.

#### Textual representation

In this study, the input from textual sentences describe the basic disease symptoms and which may imply useful information behind the texts. Chinese clinical texts are dramatically different from clinical texts in English, as there is no separator between words. At present, many words segment tools are be proposed for Chinese text analysis such as THULAC (https://github.com/thunlp/THULAC-Python), Jieba (https://pypi.org/project/jieba/). However, there are no special Chinese word segmentation tools for the clinical domain. In this study, Chinese clinical sentences are segmented into single Chinese characters. ( were segmented into 

Formally, given an input sentence $x =x_{1},\dots,x_{n}$, the BiLSTM model first finds the word or phrase embedding *e*(*x*_*i*_)∈*R*^*L*^ of each word *x*_*i*_ in the lookup table *E*∈*R*^*L*×*V*^, where *L* is the dimension of embedding vector and *V* represents the vocabulary size.

BiLSTM models a recurrent state transform sequence from an input sequence to a hidden state sequence. Basically, a LSTM represents each time step with an input, a memory and an output gate, denoted as *i*_*t*_,*f*_*t*_,*o*_*t*_, respectively.

1 $$ \begin{aligned} &i_{t} = \lambda \left(W_{xi}x_{t} + W_{hi}h_{t-1} + W_{ci}c_{t-1} + b_{i}\right) \\ &f_{t} = \lambda \left(W_{xf}x_{t} + W_{hf}h_{t-1} + W_{cf}c_{t-1} + b_{f}\right) \\ &c_{t} = f_{t} \odot c_{t-1} + i_{t} \odot tanh\left(W_{xc}x_{t}+W_{hc}h_{t-1}+b_{c}\right) \\ &o_{t} = \lambda\left(W_{xo}x_{t} + W_{ho}h_{t-1} + W_{co}c_{t} + b_{o}\right) \\ &h_{t} = o_{t}\odot tanh\left(c_{t}\right) \end{aligned}  $$

where *λ* is the element-wise sigmoid function and ⊙ is the element-wise product. *x*_*t*_ is the input vector (word embedding) at the time *t*, and *h*_*t*_ is the hidden state vector, *W* are weight matrices, and *b* are biases.

The BiLSTM has two parallel layers in both forward and backward direction. Therefore, we get a sequence $h_{ft}=\left \{\overrightarrow {h_{1}},\dots,\overrightarrow {h_{n}}\right \}$ from left to right, and another sequence $h_{bt}=\left \{\overleftarrow {h_{1}},\dots,\overleftarrow {h_{n}}\right \}$ from right to left. We then concatenate these two hidden outputs as one total output:

2 $$ h_{t} =\left[h_{f};h_{b}\right]  $$

Based on the BiLSTM modeling, we obtain textual representation *h*_*t*_.

Attention mechanism has been demonstrated success in machine learning. In this section, we use the words attention to enhance performance of the disease prediction. In EHRs, not all words or phrases are equally important for predicting positive blood culture, under the assumption that the label of *x*_*t*_ is not determined by *h*_*t*_ only. Let *h* be a matrix consisting of output vectors $[h_{1},\dots,h_{n}]$ that the BiLSTM layer producted, where *n* is the sentence length. The attention layer produces a new representation sequence $h^{(r)}=\left (h^{(r)}_{1},\dots,h^{(r)}_{n}\right)$, where $h^{(r)}_{t}$ is the representation at step *t* and can be calculated as follows:

3 $$ h^{(r)} = tanh\left(h\cdot\alpha^{T}\right)  $$

where *tanh* is the activation function, *α*_*t*_ is the weight vector for each word in the sentence calculated as follows:

4 $$ \alpha_{t} = softmax\left(w^{T}\cdot tanh(h)\right)  $$

where *softmax* is the normalization function, $h\in \mathbb {R}^{d^{w} \times T}, d^{w}$ is the dimension of word vectors, *w* is a trained parameter vector. Finally, the attention layer produces a new representation sequence $h^{(r)}=\left (h^{(r)}_{1},\dots,h^{(r)}_{n}\right)$.

#### Numerical representation

The laboratory biochemical indicators are all the numerical features in our clinical data, and some of the values are correlated. Using these values directly are not applicable. Previous work showed that the Denoising Autoencoder (DAE) network can be exploited for noise and correlation reduction, feature extraction [29, 30]. Therefore, we employ DAE to extract the numerical features.

DAE is a machine learning model that aims to reconstruct input data as close as possible. A DAE generally comprises two parts: encoder and decoder. The initial input *x* is corrupted to $\tilde x$ by a stochastic mapping $\tilde x \sim q\left (\tilde x |x\right)$. Then the encoder maps an input $\tilde x$ to a hidden representation *h*^(*z*)^ via a nonlinear transformation.

5 $$\begin{array}{@{}rcl@{}} h^{(z)} = f\left(W\tilde x + b\right) \end{array} $$

And then the decoder maps the hidden representation *h*^(*z*)^ back to reconstruct data $\tilde x$ via another nonlinear transformation:

6 $$\begin{array}{@{}rcl@{}} h^{(d)} = g\left(\hat W h^{(n)} + \hat b\right) \end{array} $$

Where *W* and *b* represent the weight and bias matrices of encoder, respectively, while $\hat W$ and $\hat b$ represent the weight and bias matrices of decoder, respectively. Moreover, *f*() and *g*() denote non-linear activation functions, such as the sigmoid function, hyperbolic tangent, and rectified linear function. Finally, we obtain a refined representation *h*^(*d*)^ of the discrete values.

#### Out layer

A fully connected layer is used to combine two types of vectors from textual representation and numerical representation. This layer can be computed as:

7 $$\begin{array}{@{}rcl@{}} h^{(A)}= g\left(W^{(A)}\right)\cdot\left[\begin{array}{c} h^{(r)} \\ h^{(d)} \end{array}\right] \end{array} $$

where *W*^(*A*)^ is a parameter, and *g* is ReLU function. Here, the dropout technique is utilized to avoid the overfitting. Finally, we employ the *softmax* activation function as the classifier in the bottom of the fully connected layer to obtain the output.

## Experiments

### Dataset construction and data preprocessing

To construct the dataset of this task, we gather a large amount of EHRs data, which is from the first affiliated hospital of Zhengzhou University, with a span of 2 years ranging from 2017 to 2018. The raw EHRs contain some personal privacy, e.g., patient’s name, hospitalization number, resident ID number etc., so we remove these information by preprocessing. In addition, all patients were at least 16 years old at the moment of admission. We selected patients who had at least one blood culture test taken during hospitalization as our goal to construct a model which can distinguish patients who has a serious illness without infections and patients with bloodstream infections. We defined two patients groups. One group consisted of patients who had positive blood culture result at least once after their admission in the hospitalization. And in another group, the results of the blood cultures tests are all negative during hospitalization. The goal of this research is to predict the risk of bloodstream infection of patients during the hospitalization by predicting positive blood cultures. We did not distinguish the pathogen types. As such, we cannot rule out that positive blood cultures, which may be the result of false positive predictions caused by skin contaminants. Finally, we select a set of patients from the EHRs based on the following criteria:

- A patient who had blood culture positive results during hospitalization is selected as positive example.
- A patient whose results of all blood culture tests are negative during hospitalization is selected as negative example.

Based on the above steps, we get total 28043 patients, in which 25056 patients are negative examples and 2987 patients are positive examples. This is an extremely imbalance dataset, and is problematic for directly conducting the experiments. To tackle this problem, we employ undersampling method to balance the classes. Specifically, we randomly deletes the majority-class data for balancing the dataset. At last, there are total 5963 examples in the dataset after undersampling. For the purpose of fully utilizing these dataset, we repeated the random undersampling for ten times to get the average prediction results.

The extracted data consists of textual description and numerical indicators in EHRs. The textual information contain the patient’s CC and AR in EHRs, which includes the patient’s disease symptoms and test results. These information is important and closely related to the patient’s health. In addition, we extract the laboratory numerical parameters in EHRs. First, we removed clinical parameters which present in a small fraction of patients. The remaining parameters were further grouped in 27 parameter features as illustrated in Table 1, including three basic features (sex, age and temperature) and twenty-four blood test indexes. After removals of missing data, we further normalize the data:

8 $$\begin{array}{@{}rcl@{}} \tilde{x} = \frac{x-avg}{std} \end{array} $$

**Table 1:** Overview of included clinical characteristics of patients

**Number**	**Clinical parameters**	**Abbreviation**
F1	Sex	SEX
F2	Age	AGE
F3	Temperature [ ^∘^C]	TEMP
F4	C-reactive protein concentration	CRP
F5	Procalcitonin	PCT
F6	Prothrombine time	PT
F7	Prothrombin time activity	PT%
F8	Thrombin time	TT
F9	Activated partial thromboplastin time	APTT
F10	Fibrinogen degradation products	FDP
F11	Fibrinogen	FIB
F12	D-Dimer	D-Dimer
F13	White blood cell	WBC
F14	Neutrophil	NEUT
F15	Blood platelet	PLT
F16	Red blood cell	RBC
F17	Hemoglobin	HB
F18	Platelet	PLT
F19	Neutrophil count	NEUT#
F20	Neutrophil ratio	NEUT%
F21	Lymphocyte count	LYMPH#
F22	Lymphocyte ratio	LYMPH%
F23	Hematocrit	HCT
F24	Red cell distribution width	RDW
F25	Mean platelet volume	MPV
F26	Basophil ration	BASO%
F27	Thrombocytocrit	Pct

where *x* is the value, *avg* the average of all values and *std* the standard deviation.

The textual and basic features were extracted from admissions records after admission. Normally, patients may have multiple blood tests during hospitalization, but in this study we only analyzed blood test indexes before the first positive blood culture test. For the positive group of patients, we extracted the test results before the first blood culture test was positive, including the maximum PCT and CRP values and the seven terms of coagulation test results with the maximum PT value. Besides, we also extracted the latest 15 blood test indexes before the first positive blood culture. For the negative group of patients, we extracted the minimum PCT and CRP values during hospitalization. The latest another 25 clinical indexes were extracted before the first blood culture test.

In summary, we obtain one dataset across 5963 patients with each one containing 27 clinical features. A patient was labeled as ‘1’ with a positive blood culture and ‘0’ otherwise.

### Evaluation metric

In our study, we use widely-used evaluation measures to evaluate the performance of prediction models, including precise, recall and F-measure. These measures can be defined by True Positives (TP), False Positives (FP), False Negatives (FN) and True Negatives (TN) in Table 2. Here TP, FN, FP and TN are the number of examples correctly labeled as positive, the number of positive examples incorrectly labeled as negative, the number of negative incorrectly labeled as positive, the number of negative examples correctly labeled as negative, respectively.

**Table 2:** Four kinds of prediction results

	**Predict as positive**	**Predict as negative**
**Positive examples**	TP	FN
**Negative examples**	FP	TN

The recall rate denotes the ratio of the number of positive examples that are correctly classified as positive to the total number of positive examples. This measure is very important for our task, because prediction models intend to find out positive examples as much as possible. The precision of a model denotes the ratio of the number of positive examples that are correctly classified as positive to the number of examples that are classified as positive. The prediction precision evaluates the correct degree of prediction model, which are defined as :

9 $$\begin{array}{@{}rcl@{}} recall= \frac{TP}{TP + FN} \end{array} $$

10 $$\begin{array}{@{}rcl@{}} precision = \frac{TP}{TP + FP} \end{array} $$

Obviously, a good prediction model desires to get high value of recall rate and precision. However, there exists trade-off between the recall rate and precision. Therefore, a comprehensive measure of recall rate and precision is necessary. F-measure is the harmonic mean of recall rate and precision, which is defined as:

11 $$\begin{array}{@{}rcl@{}} F-measure = \frac{(1+\alpha) \times recall \times precision}{recall + \alpha \times precision} \end{array} $$

where *α*∈(0,+*∞*), is the weight of recall metric. In this research, we use *α*=1.

All the above evaluation measures range from 0 to 1. Obviously, an ideal prediction model should hold high values of recall rate and F-measure. In the experiment, we evaluate the performances of models in terms of recall and F-measure. We also get the precision results since it has been included in the comprehensive F-measure.

### Experimental settings

For datasets, we performed 10-fold cross-validation following prior work [31]. The whole dataset is split into ten sections, each decoded by the model trained from the remaining nine sections. We randomly choose one section from the nine training sections as the validation dataset to tune the model parameters.

In our experiments, there are two types of parameters, containing model hyper-parameters and other setting. Typically, *L* denotes the dimension of the word vectors, *L*_*bilstm*_ is the maximum length of the input textual sequences, *N*_*AE*_ is the number of Autoencoder layer, *N*_*MLP*_ is the number of fully connected layers. The dropout rate in fully connected layer is denoted as *R*_*dropoout*_. *λ* is the initial learning rate for AdamGrad. In our model, the word embedding *E*, is randomly initialized with uniform samples from $[-\sqrt {\frac {6}{r+c}},+\sqrt {\frac {6}{r+c}}]$, where *r* and *c* are the number of rows and columns in the structure. Parameters are shown in Table 3.

**Table 3:** Parameters of our model in the experiment

**Type**	**Parameters**
Training	*λ*=0.001,*epochs*=20
	*batchsize*=16
Embedding	*dim*(*emb*(*L*))=100
	*epochs*=20
BiLSTM	*L*_*bilstm*_=128
	*R*_*dropoout*_=0.5
AutoEncoder	*N*_*AE*_=6,*N*_*MLP*_=2

To validate the effectiveness of the proposed approach for prediction of blood culture outcome, we compared our approach with the several representative methods, four discrete models including Logistic Regression (LR), Naive Bayes (NB), Support Vector Machine (SVM) and Adaboost Decision Tree (ADT). These discrete models have been extensively used for classification tasks, giving competitive results. And three neural models: Convolutional Neural Network (CNN), Bi-directional Long Short Term Memory (BiLSTM) and a hybrid neural network models which integrates the Autoencoder (AE) with the ABiLSTM to make use of two types of features. And all above baseline models are implemented with Sklearn and Tensorflow.

We design three experiments to evaluate our approach: (1) Only laboratory biochemical indicators as input to the model (Numerical input only, noted as Numerical). (2) Only the textual features such as patient’s information, symptoms and admissions records in the EHRs as input to the model (noted as Textual). (3) Numerical and textual features as input to the model.

## Results

In this section, we evaluate the performance of our hybrid model based on the ability to accurately predict the outcome of a blood culture test. Firstly, we compare the ability of the different methods to predict positive blood culture based on the laboratory features. Based on the constructed dataset, Table 4 shows experimental results of different methods. We can know that the LR model proposed by Chen [32] only gives 78.91% F-measure. The main reason is that this model only take laboratory indicators as input, ignoring the textual description information from EHRs. This limits the performance of the task. The NB and SVM models gives 81.95% and 83.23% F-measure, outperforming the LR model. This shows the effectiveness of these two models in this task. Among all models, ADT gives the relatively highest results, giving 85.56% F-measure. The main reason is that ADT is a boosting model which contains multiple meta classifiers and uses the assembling mechanism, and this makes ADT model more powerful.

**Table 4:** Experimental results of numberical features

**Model**	**Precision (%)**	**Recall (%)**	**F-measure (%)**
LR	78.56	79.26	78.91
NB	80.24	83.73	81.95
SVM	84.56	81.95	83.23
ADT	84.64	86.51	85.56
AVG	82.00	82.86	82.41

Table 5 shows the experimental results of different methods based on textual description information from EHRs. Among all models, the neural network models get the relatively good results. The BiLSTM gives 72.59% F-measure and the ABiLSTM could get 73.21%. This demonstrates that the neural network has powerful ability to fully learn the intrinsic features from the textual description. However, we can easily observe that using only textual information, no better than laboratory indicators features.

**Table 5:** Experimental results of textual features

**Model**	**Precision (%)**	**Recall (%)**	**F-measure (%)**
LR	66.21	59.31	62.57
NB	59.25	62.75	60.95
SVM	63.56	65.25	64.39
ADT	65.54	67.25	66.38
CNN	69.21	71.18	70.18
BiLSTM	76.35	69.19	72.59
ABiLSTM	75.35	71.19	73.21
AVG	66.92	55.01	67.18

The experimental results of different methods based on laboratory+textual features are shown in Table 6. By integrating DAE, ABiLSTM+DAE could achieve 91.23% F-measure on laboratory+textual features, which is significantly higher than other methods. Remarkably, we can know that all models can get better performance based on the combination of numerical and textual features compared to the only laboratory (numerical) and textual features. This is because different types of features in EHRs can both give their own contributions. Meanwhile, the results from only laboratory features are better than that from numerical features.

**Table 6:** Experimental results of hybrid numberical and textual features

**Model**	**Precision (%)**	**Recall (%)**	**F-measure (%)**
LR	83.55	85.23	84.38
NB	84.36	87.68	85.99
SVM	86.12	87.41	86.76
ADT	85.72	87.39	86.55
CNN	87.36	88.69	88.01
BiLSTM	87.16	90.01	88.56
CNN+DAE	89.21	90.36	90.01
BiLSTM+DAE	90.32	91.59	90.96
ABiLSTM+DAE	90.15	92.35	91.23
AVG	87.11	89.68	88.05

## Discussion

In this section, we analyze the results on constructed test set to show the main reasons that the hybrid model (ABiLSTM+DAE) is better than the discrete models (ADT). We characterize the main errors generated by the hybrid model. Table 7 shows the number of positive examples for correct/incorrect recognition. For positive blood culture prediction, the number of examples that were addressed correctly by ABiLSTM+DAE model but incorrectly by the ADT model is over 3.5 times compared to those addressed by the ADT model correctly but by the ABiLSTM+DAE model incorrectly (345 versus 97). Moreover, among the 345 examples that were addressed correctly ABiLSTM+DAE model but incorrectly by the ADT model. This indicates that the hybrid model helps to capture more features information to improve prediction performance.

**Table 7:** Comparisons between the ABiLSTM+DAE and ADT on the test set

**Model**		**Recongition(%)**
BiLSTM+DAE	ADT	
Correct	Correct	1398 (49%)
Correct	Wrong	345 (12%)
Wrong	Correct	97 (3.3%)
Wrong	Wrong	107 (3.6%)

## Conclusion

It is challenging to predict patients at risk for bloodstream infection based on laboratory test results and the clinical profile of the patient. Therefore, the ability to accurately predict a positive outcome of blood cultures at an early stage may save lives and make full use of medical resources. In this paper, we propose a hybrid neural networks model by integrating the attention based BiLSTM and denoising Autoencoder networks to predict the outcome of a blood cultures. Based on the constructed dataset from the raw Chinese EHRs, experimental results show that this model can accurately determine the outcome of blood culture test at the moment the blood sample was taken. In this study, we only used the contents of chief complaints and admissions records in EHRs and did not integrate all contents of EHRs into the model, such as medical orders, surgical records, nursing records and so on. Therefore, future research will focus on how to integrate different types of medical information to improve the prediction effect for positive blood culture.

## Data Availability

The experimental data will not be shared as it involved in privacy conditions.

## Abbreviations

EHRs: Electronic Health Records
BCs: Blood Cultures
CC: Chief Complaints
AR: Admissions Records
ABiLSTM: Attention-based Bi-directional Long Short Term Memory
DAE: Denoising Autoencoder
TP: True Positives
FP: False Positives
FN: False Negatives
TN: True Negatives
LR: Logistic Regression
NB: Naive Bayes
SVM: Support Vector Machine
ADT: Adaboost Decision Tree
CNN: Convolutional Neural Network
AE: Autoencoder
